# Intestinal Disorder in Zebrafish Larvae (*Danio rerio*): The Protective Action of N-Palmitoylethanolamide-oxazoline

**DOI:** 10.3390/life12010125

**Published:** 2022-01-16

**Authors:** Davide Di Paola, Sabrina Natale, Carmelo Iaria, Marika Cordaro, Rosalia Crupi, Rosalba Siracusa, Ramona D’Amico, Roberta Fusco, Daniela Impellizzeri, Salvatore Cuzzocrea, Nunziacarla Spanò, Enrico Gugliandolo, Alessio Filippo Peritore

**Affiliations:** 1 Department of Chemical, Biological, Pharmaceutical, and Environmental Science, University of Messina, 98166 Messina, Italy; davide.dipaola@unime.it (D.D.P.); sabrina.natale@unime.it (S.N.); carmelo.iaria@unime.it (C.I.); rsiracusa@unime.it (R.S.); rdamico@unime.it (R.D.); rfusco@unime.it (R.F.); dimpellizzeri@unime.it (D.I.); aperitore@unime.it (A.F.P.); 2 Department of Biomedical and Dental Sciences and Morphofunctional Imaging, University of Messina, 98166 Messina, Italy; cordarom@unime.it; 3 Department of Veterinary Science, University of Messina, 98166 Messina, Italy; rcrupi@unime.it (R.C.); egugliandolo@unime.it (E.G.); 4 Department of Pharmacological and Physiological Science, Saint Louis University School of Medicine, Saint Louis, MO 63104, USA

**Keywords:** PEAOXA, inflammation, DSS

## Abstract

IBD (Inflammatory Bowel Disease) is an inflammatory disease affecting the gastrointestinal tract that is common in both humans and veterinarians. Several studies have revealed the pharmacological properties of the oxazoline of palmitoylethanolamide (PEAOXA). Zebrafish larvae were exposed to sodium dextran sulphate (DSS) to induce enterocolitis and study the protective action of PEAOXA. After repetitive exposure with 0.25% DSS, larvae presented gut alteration with an increase in mucus production. Furthermore, DSS exposure induced an increase in the inflammatory pathway in the intestine, related to an increase in the Endoplasmic-reticulum (ER) stress genes. PEAOXA exposure at a concentration of 10 mg/L decreased the DSS-induced gut damage and mucus production, as well as being able to reduce the inflammatory and ER stress-related genes expression. In conclusion, our results demonstrate that the alterations induced by repeated exposure to DSS were counteracted by PEAOXA action that was able to inhibit the increase in inflammation and ER stress involved in the progression of enterocolitis.

## 1. Introduction

The inflammatory response, when properly regulated, helps to maintain cellular homeostasis and resistance to disease, but its persistence, i.e., chronic inflammation, can lead to autoimmune disorders related to various diseases, such as diabetes, neurodegenerative diseases, asthma or arthritis [1].

While murine inflammatory bowel disease (IBD) models are useful for elucidating disease pathogenesis they are prohibitively expensive to use for in vivo drug screening. Zebrafish embryos were previously assessed as models of chemically-induced enterocolitis and IBD and potentially provide a low-cost animal model of acute colitis for anti-inflammatory drug development programs in the early phases [2,3]. Zebrafish use the proximal intestine (PI) for food storage and mixing, as they lack a stomach [4,5]. The middle intestine (MI) of the zebrafish is composed of enterocytes, which are responsible for nutrient absorption, and goblet cells that produce mucin. Moreover, the distal portion of the MI contains some specialised enterocytes that are presumed to play a role in intestinal immunity [4]. While the architecture of the short distal intestine (DI) suggests a role similar to that of the colon in mammals [5,6]. Inflammatory bowel disease is a chronic, relapse-prone disease of rising worldwide prevalence [7]. Flare-ups of IBD are treated symptomatically with anti-inflammatory medicine because the condition is now incurable. Because of the considerable adverse effects of long-term medication administration and the proportion of non-responders, current therapeutic methods for IBD remain unsatisfactory [8,9].

The two most frequently used chemical models require administration of sodium dextran sulphate (DSS) via drinking water or intrarectal administration of trinitrobenzene sulfonic acid (TNBS). Ingestion of DSS leads to colonic cell death and inflammation as a result of colonic toxin activity. Several studies have shown that repetitive exposure to DSS early in zebrafish development generated a phenotype of marked mucus production with no observed changes in the number of goblet cells in the midgut and oesophagus and overproduction of pro-inflammatory cytokines [10].

N-palmitoylethanolamine (PEA) is an endogenous lipid that plays a role in maintaining cellular homeostasis by acting as a resolution mediator of inflammatory processes. Several studies have shown the pharmacological properties of a new formulation of PEA, the oxazoline of PEA (2-pentadecyl-2-oxazoline or PEAOXA), which shows anti-inflammatory and especially analgesic actions in different models both in vitro and in vivo [11]. Moreover, PEAOXA was identified in natural compounds such as coffee [12]. The protective action of PEAOXA at the intestinal level was demonstrated through previous studies. In fact, PEAOXA treatment showed ameliorations on behavioural deficits induced by colon inflammation with the involvement of the gut–brain axis [13]. The neuroprotective action of PEAOXA not only leads to a decrease in alterations and damage associated with inflammation but is also reflected in improvements in the behavioural sphere. In fact, PEAOXA was shown to improve depressive-like and anxiety-associated behaviours in a mouse model of neuropathic pain [14].

In this study, sodium dextran sulphate (DSS) was applied on zebrafish larvae to induce enterocolitis and assess the protective effect of PEAOXA. The expression levels of inflammatory mediators were determined to explore the anti-inflammatory mechanism of PEAOXA.

## 2. Materials and Methods

### 2.1. Zebrafish Maintenance and Embryo Collection

Wild-type (WT) mature zebrafish at an age of 6 months were used for embryo production. Zebrafish Maintenance and Embryo Collection of fertilised eggs were provided from the Center of Experimental Fish Pathology (Centro di ittiopatologia Sperimentale della Sicilia, CISS), University of Messina, Italy. The fish were fed both with dry and live food twice a day at 3% of body weight (BW). For a successful reproduction, mature females and males were mated at 2:1 ratio. The day after, the eggs were collected, bleached and afterward non-fertilised eggs were discarded. Fertilized eggs were transferred into 24-well plates with test solutions and incubated at 28 °C at a 14:10 h day/night light regime. Only embryos that reached the blastula stage were used for experiments. Fish Embryo Toxicity (FET) test was performed according to OECD [15] and ISO 15088.

### 2.2. Repeated DSS Injury Model

Zebrafish embryos were exposed to PEAOXA for 24–120 h post-fertilization (hpf) to measure the protective effects over a continuing observation period. As we described in previous paper [16], at 4 hpf healthy embryos were placed in 24-well culture plates (1 embryo in 2 mL solution/well). PEA-OXA was dissolved for stock solution as previously described [17] in methanol 0.05%, and after PEAOXA dissolved in the stock solution was after diluted with Embryo medium (15 mM NaCl, 0.5 mM KCl, 1 mM CaCl_2_, 1 mM MgSO_4_, 0.15 mM KH_2_PO_4_, 0.05 mM Na_2_HPO_4_, 0.7 mM NaHCO_3_ a pH 7.3) to the tested different doses (3 and 10 mg/L). Each group had 3 replicate wells. Each experiment was replicated three times. Embryos were photographed under a stereomicroscope (Leica M0205C, Multifocus, Wetzlar, Germany) during the exposure period, and the percentage of aberrant embryos was counted every 24 h. A total of 24 (hpf) larvae were put in freshly prepared 0.25% (*w*/*v*) colitis grade DSS (36,000–50,000 MW, MP Biomedical) for 4 days to induce intestinal damage. In the PEA-OXA group, larvae were exposed to 3 mg/L and 10 mg/L of PEA-OXA respectively, for all the time of DSS exposure. The PEAOXA solution to be exposed to zebrafish embryos/larvae was freshly changed every 8 h from 4 hpf to the end of the experiment to decrease any solubility issues and precipitate. Larvae were not fed for the duration of the DSS treatment experiments 120 hpf. During the exposure time, the embryonic development and various mortality rates were observed.

### 2.3. Histopathology

At the end of the 120 hpf, larvae were processed by transferring them through a dilution series of ethanol, with an increasing amount of alcohol, to dehydrate the tissue, and after replaced in the alcohol in order for the paraffin to infiltrate the tissue. The sections from each group were stained with haematoxylin and eosin (H&E) and Periodic acid Schiff stain (PAS) for quantification of goblet cells as previously described [18,19]. A scoring system (0–3) was used to examine the effect of PEAOXA on DSS-treated fish, with parameters such as an increase in the number of goblet cells, enlargement of the intestinal wall, and poor epithelial integrity being taken into account. Alcian blue (Sigma-Aldrich) binding assay was performed according to what was seen previously [20]. Paraffined slides of larvae were stained following the Alcian Blue bio-optica protocol.

### 2.4. Real-Time PCR

The total RNA from larval zebrafish was isolated by the TRIzol reagent (Invitrogen, Waltham, MA, USA) with a homogenizer. Total RNA was isolated according to the manufacturer’s instructions. The ratio of absorbance at 260–280 nm, as well as the banding patterns on a 1% agarose formaldehyde gel, were used to verify the quality of the RNA in each sample. RNA quality was evaluated by gel electrophoresis, with the concentration measured with NanoDrop 2000 (Thermo Scientific, Waltham, MA, USA). iScript RT-PCR kit (Bio-Rad, Hercules, CA, USA) was used to synthesize first-strand cDNA according to manufacturer’s recommendations. Briefly, the reverse transcription master mix was prepared adding 1 μg of RNA template the iScript RT Supermix (5 × RT supermix with RNase H+ Moloney (gray cap, 25 or 100 reactions) murine leukemia virus (MMLV) reverse transcriptase, RNase inhibitor, dNTPs, oligo(dT), random primers, buffer, MgCl_2_ and stabilizers) and the nuclease-free water. The complete reaction mix was incubated in a thermal cycler (Priming 5 min at 25 °C, Reverse transcription 20 min at 46 °C, RT inactivation for 1 min at 95 °C). A portion of 1 μL of total RT products was used directly for real-time PCR. PCR analysis by SYBR Green method on a StepOnePlus Real-Time PCR System (Applied Biosystems, Foster City, CA, United States). PCR conditions were initial denaturation at 95 °C for 15 min, followed by 45 cycles of amplification at 95 °C for 20 s and 60 °C for 40 s. Final extension at 60 °C for 60 s and hold at 4 °C was then performed. Table 1 showed the detailed information on the primers of β-actin, inflammatory-related genes and ER-stress related genes as previously reported [21,22]. β-actin transcript was used as a housekeeping gene [23,24,25]. The main PCR protocol was referenced in our previous study and described [26]. The relative transcriptional levels of each gene were estimated based on past research [27]. Data analysis was performed using the 2^−∆∆Ct^ method and the results are expressed as fold-changes.

### 2.5. Statistical Evaluation

All values in the figures and text are expressed as the mean ± standard error (SE) of N number of experiments. The results were analysed by two-way ANOVA followed by a Tukey post-hoc test for multiple comparisons. The data were tested for average distribution with the Kolmogorov–Smirnov test (*p* < 0.05) and they were represented as mean ± standard deviation (SE), (alpha value of 0.05). Statistical analysis was performed using GraphPad Prism 8.

## 3. Results

### 3.1. PEAOXA Effect on DSS-Induced Intestinal Alteration

**Figure 1:**
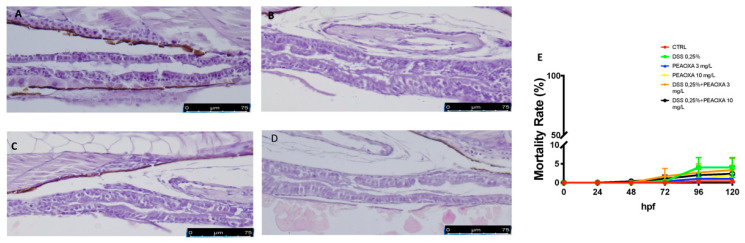
PEAOXA effect on DSS-induced intestinal alteration. Longitudinal sections of the mid-distal intestinal junction were stained with haematoxylin and eosin (H&E); (**A**) CTRL, (**B**) DSS, (**C**) DSS+PEAOXA 3 mg/L; (**D**) DSS+PEAOXA 10 mg/L. (**E**) Mortality rate.

The histopathological examination of the zebrafish intestinal sections after H&E staining was performed to assess the protective effect of PEAOXA on the mild intestine morphology after DSS treatment. Zebrafish repetitively exposed to DSS caused tissue inflammation as well as major pathological alterations such as severe epithelium damage and an increase in the number of goblet cells (Figure 1). Fish treated with PEAOXA showed a greater protective effect against the DSS-induced alteration, resulting in an overall decrease of histological damage in DSS (Figure 1). Similar to the DSS lesion in mice, DSS-induced enterocolitis also caused zebrafish gut alterations (Figure 1). No abnormalities and only a slight but not significant mortality was caused at 120 hpf for all the groups (CTRL, DSS 0.25%, PEAOXA 3 and 10 mg/L, DSS 0.25% + PEAOXA 3 or 10 mg/L) Figure 1. 

### 3.2. DSS-Induced Lysosomal Acidification, Mucin Loss and Intestinal Alteration

**Figure 2:**
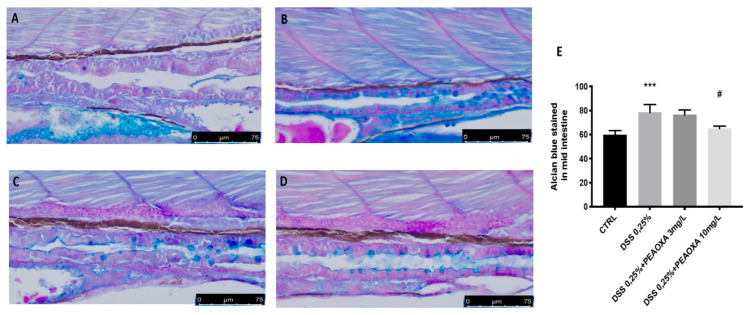
PEAOXA effect on DSS-induced mucopolysaccharides production. Whole-mount control and DSS-exposed larvae stained with Alcian blue. Longitudinal sections of the mid-distal intestinal junction from control and DSS-exposed larvae, stained with Alcian blue; (**A**) CTRL, (**B**) DSS; (**C**) DSS+PEAOXA 3 mg/L; (**D**) DSS+PEAOXA 10 mg/L. Increase mucus production (blue staining) (**E**). Values represent the mean ± SE *** *p* < 0.001 compared with CTRL at the same time point and # *p* < 0.05 relative to DSS 0.1% group.

Alcian Blue staining, which assesses epithelial mucus production, revealed that the amount of mucus produced was normal, with no alterations in the number of goblet cells in the control group with no DSS immersion. DSS repeated exposure resulted in decreased mucus production (blue staining) but did not show an effect on goblet cells (Figure 2). PEAOXA pre-treatment, at a dose of 10 mg/L, but not at a 3 mg/L, showed protection on mucus production in the intestine of larvae repeatedly immersed in DSS (Figure 2). Like the DSS lesion in mice, DSS-induced enterocolitis also causes zebrafish gut alterations (Figure 2). 

### 3.3. Effect of PEAOXA on mRNA Expression Levels of Inflammatory Pathway

**Figure 3:**
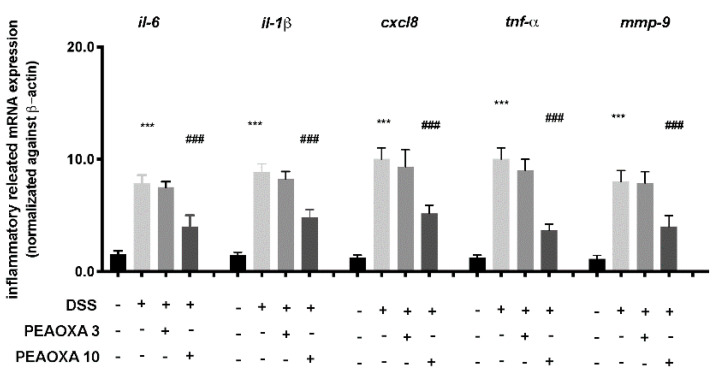
Effect of PEAOXA on mRNA expression of cytokine genes along the zebrafish intestine in presence (+) or absence (-) of DSS and PEAOXA 3 or 10 mg/L exposure. Expression values are normalised against the expression of β-actin mRNA, which was not significantly different between any of the treatment groups. Values represent the mean ± SE *** *p* < 0.001 compared with CTRL at the same time point and ### *p* < 0.001 relative to DSS 0.1% group.

To investigate the anti-inflammatory activity of PEAOXA, we analysed the expression of several cytokines implicated in intestinal immunity. Our analysis revealed a general increase in the expression of IL-6, interleukin-1β (IL-1β), IL-8 (cxcl8), TNF-α and Mmp-9 after DSS repetitive exposure in zebrafish larvae (Figure 3). PEAOXA exposure on larvae was able to reduce the mRNA levels of all the inflammatory-related genes compared to the DSS group. Contrarily, no effect was seen for the PEAOXA 3 mg/L pre-treatment group compared to the DSS group (Figure 3).

### 3.4. Effect of PEAOXA on mRNA Expression Levels of ER Pathway

**Figure 4:**
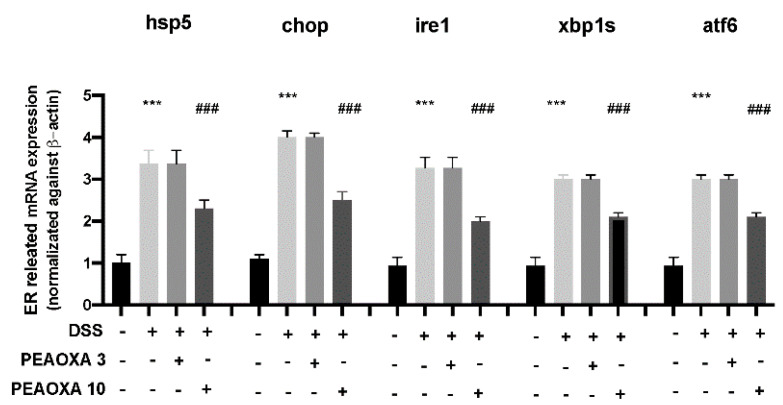
Effect of PEAOXA on mRNA expression of ER-related genes (hspa5, chop, ire1, xbp1s, and atf6) along the zebrafish intestine presence (+) or absence (-) of DSS and PEAOXA 3 or 10 mg/L exposure. Expression values are normalised against the expression of β-actin mRNA, which was not significantly different between any of the treatment groups. Values represent the mean ± SE *** *p* < 0.001 compared with CTRL; ### *p* < 0.001 relative to DSS 0.1% group.

To investigate the protective effect on the mild intestine of PEAOXA, we analysed the expression of ER stress-related genes. Our analysis revealed a general increase in the expression of hspa5, chop, ire1, xbp1s, and atf6 after DSS repetitive exposure in zebrafish larvae (Figure 4). The low dose of PEAOXA did not show an effect on the ER stress pathway. PEAOXA exposure at 10 mg/L was able to reduce the ER stress in the mild intestine of zebrafish larvae. In fact, PEAOXA decreased hspa5, chop, ire1, xbp1s, and atf6 overexpression after DSS repetitive exposure (Figure 4).

## 4. Discussion

Among the most used and important vertebrate animal models, there is certainly the zebrafish, because of the many advantages it offers over cellular and other animal models it is widely used in pharmacology studies [28]. In the current study, we assessed the effect of PEAOXA in an enterocolitis model DSS-induced on zebrafish embryos. Zebrafish models of IBD provide substantive advantages in modelling repetitive intestinal injury and repair. In this regard, most IBD models have used DSS, which results in epithelial injury and inflammation. DSS damage was reported to result from the formation of intraluminal fatty acid complexes resulting in decreased barrier function during DSS treatment [29,30]. Chronic models of DSS in mice have been reported, but take months to develop [31]. In zebrafish models, increased mortality, reduced mucus formation, and impaired autophagy have all been documented in response to recurrent DSS damage, all within 2 weeks; although the inflammatory response is prevalent in the intestine, extraintestinal consequences may occur due to the route of delivery. The tractability of the DSS-induced zebrafish enterocolitis models for finding disease-modifying drugs was investigated using a library of well-characterised chemicals and was demonstrated by the detection of known antibiotics and anti-inflammatory compounds. The enterocolitis DSS-induced model shows a curious feature when compared to the different animal models, in fact, alterations in mucus physiology reveal a dramatic variation in phenotypes, with adult mice having a modest number of goblet cells and an increase in the number of goblet cells or mucus accumulation observed in zebrafish larvae exposed to respectively high doses of DSS. Our data show that DSS causes gut alterations in zebrafish, such as alterations of the intestinal epithelium, accompanied with an increased mucus production and enlarged goblet cells. PEAOXA treatment at a dose of 10 mg/L showed a protective action on alteration of intestinal epithelium after DSS-induced enterocolitis. Moreover, PEAOXA protective action was seen on the goblet cells formation in the mid intestine and in the mucus reduction of larvae, in fact, DSS-induced increase in mucus production in the intestine of larvae was reverted by PEAOXA exposure, suggesting a preserving action on the DSS-induced alteration structure. The intestinal mucus layer is an important structural barrier that is disrupted in IBD because intestinal mucus is known to be an important scaffold for antimicrobial activity in the mammalian intestine [32]. PEAOXA treatment showed a protective role in the intestinal structure in the early stage of zebrafish development. The immune system is the basis of the interaction between the host and microbes. The immune system is important to understand the interactions between host and microbes. Moreover, in zebrafish, the immune system is very similar to humans. The adaptive immunity of zebrafish develops after only 4 weeks, within which time zebrafish defend themselves only by innate immunity. The study in the early life stages of zebrafish, therefore, allows the assessment of the innate defence mechanism without the involvement of adaptive immunity [33]. The enrichment of endocytic epithelial cells, leukocytes, and antimicrobial gene expression implicate the midgut as an immunologically important section of the intestine [4,34].

Proinflammatory cytokines are recognised as key players in the initiation and development of IBD, and amongst the most important are IL-1β, IL-6 and TNF-α. These chemicals were linked to both major clinical types of IBD—Crohn’s disease and ulcerative colitis—and are not limited to a single T-helper cell subgroup [35,36]. Transcript levels of IL-6, IL-1β, and TNF-α were considerably higher in both colitis groups in this investigation. Previous zebrafish studies have found an increase in IL-1β and TNF-α gene expression in adult fish a few hours after exposure to oxazolone [2] or TNBS [37]. The neutrophil-activating chemokine CxCL8 (IL-8) was significantly up-regulated following DSS exposure, confirming previous zebrafish-inflammatory investigations [3,37,38,39], albeit at a noticeably higher level than in the TNBS group. Cxcl8 regulates neutrophil infiltration to the site of inflammation, and gene transcripts were found in intestinal cells of larval zebrafish in a TNBS model [38]. Matrix metalloproteinases (MMPs), a family of degradative enzymes, are crucial factors in inflammatory conditions in humans [40]. MMP-9 is the most important protease in relation to colitis, and it is significantly overexpressed in both human and animal models of IBD [41,42,43]. In line with the results obtained in this study, Oehlers et al. reported a significant increase in MMP-9 in larval zebrafish exposed to TNBS [3]. Exposure to PEAOXA decreased the expression of pro-inflammatory cytokines increased as a result of DSS action, suggesting a protective action against intestinal alterations by decreasing the production of mediators involved in the inflammatory pathway.

The detection of increased transcription of inflammatory-related genes in the midgut suggests a molecular mechanistic immune response upon DSS-induced damage in the zebrafish gut. Furthermore, our examination of cytokine expression in the zebrafish intestine suggested a protective role of PEAOXA on conserved cellular immune homeostasis. Defects in the ER stress response and autophagy lead to intestinal inflammation [44]. Components of the ER stress response in the epithelial intestinal epithelium were documented in IBD patients and in mouse models [45,46]. Abnormal intestinal morphologies include decreased epithelial proliferation, apoptosis of epithelial cells and goblet cells, severe inflammation, and disruption of the normal balance between bacteria and the gut [47]. In the case of unfolded proteins, the accumulation of ER activates the unfolded protein response (UPR) to reestablish normal ER functions and to help the cells adapt to the environmental changes [48]. However, UPR can also induce cell death when ERS is prolonged and severe [49]. Three signaling pathways can activate UPR: the inositol requiring enzyme 1 (IRE1)-X-box binding protein 1 (XBP1) pathway, the protein kinase RNA-activated-like ER kinase (PERK)-eukaryotic translation initiation factor 2 alpha (eIF2a) pathway and the activating transcription factor 6 (ATF6) pathway [50]. Hspa5 is a regulator of the ER homeostasis activated in case of accumulation of misfolded and unfolded proteins in ER [51]. Chop is a regulator of the apoptosis pathway activated by ERS [52], while ire1 is responsible of the activation of the endoribonuclease activity that activates the xbp1s transcription factor. The mRNA expression of hspa5, chop, ire1, xbp1s, and atf6 were found to increase in zebrafish embryos indicating that DSS exposure induces ERS through the IRE1-XBP1 and ATF6 pathways. PEAOXA exposure showed a protective action on the mild intestine, decreasing the overexpression of the ER-related gene after DSS exposure, suggesting an anti-inflammatory action in the intestine of zebrafish, not only directed to the cytokines production but also on the ER stress pathway.

## 5. Conclusions

In conclusion, our results showed that in an intestinal inflammation model, exposure with PEAOXA at a dose of 10 mg/L was able to reduce the degree of intestinal damage in the DSS-induced enterocolitis model on zebrafish larvae. In addition, the anti-inflammatory activity of PEAOXA on the intestine reduced the expression of inflammatory-related genes and the expression of ER stress genes, often involved in IBD.

## Figures and Tables

**Table 1:** Primers for real-time PCR.

Gene	Primer Orientation	Nucleotide Sequence
b-actin	forward	5′-AGAGCTATGAGCTGCCTGACG-3′
	reverse	5′-CCGCAAGATTCCATACCCA-3′
Inflammatory pathway genes		
Il-6	forward	5′-AGACCGCTGCCTGTCTAAAA-3′
	reverse	5′-CAACTTCTCCAGCGTGATGA-3′
cxcl8	forward	5′-TGTTTTCCTGGCATTTCTGACC-3′
	reverse	5′-TTTACAGTGTGGGCTTGGAGGG-3′
Il-1beta	forward	5′-ATCAAACCCCAATCCACAGAGT-3′
	reverse	5′-*GGCACTGAAGACACCACGTT*-3′
tnfα	forward	5′-GCGCTTTTCTGAATCCTACG-3′
	reverse	5′-AAGTGCTGTGGtTCGTGTCTG -3′
Mmp-9	forward	5′-ACAGGGAGACGCTCATTTTG-3′
	reverse	5′-TGTTCCCTCAAACAGGAAGG -3′
Endoplasmic-reticulum-stress related genes		
hspa5	forward	5′-CAGATCTGGCCAAAATGCGG-3′
	reverse	5′-GGAACAAGTCCATGTTGAGC-3′
chop	forward	5′-CACAGACCCTGAATCAGAAG-3′
	reverse	5′-CCACGTGTCTTTTATCTCCC-3′
ire1	forward	5′-TGACGTGGTGGAAGTTGGTA-3′
	reverse	5′-ACGGATCACACATTGGGATGTT-3′
xbp1s	forward	5′-CAAAGGAGCAGGTTCAGGTAC-3′
	reverse	5′-GGAGATCAGACTCAGAGTCTG-3′
atf6	forward	5′-CATGGTGACCACAGGAGATG-3′
	reverse	5′-AAAGGAGGACATTTGAGCAG-3′

## Data Availability

The data presented in this study are available on request from the corresponding author.
